# Spore-Forming *Clostridium* (*Clostridioides*) *difficile* in Wastewater Treatment Plants in Western Australia

**DOI:** 10.1128/spectrum.03582-22

**Published:** 2022-12-08

**Authors:** Jessica M. Chisholm, Papanin Putsathit, Thomas V. Riley, Su-Chen Lim

**Affiliations:** a School of Biomedical Sciences, The University of Western Australia, Nedlands, Western Australia, Australia; b School of Medical and Health Sciences, Edith Cowan University, Joondalup, Western Australia, Australia; c Biosecurity and One Health Research Centre, Harry Butler Institute, Murdoch University, Murdoch, Western Australia, Australia; d PathWest Laboratory Medicine, Department of Microbiology, Nedlands, Western Australia, Australia; Paris-Saclay University

**Keywords:** *Clostridium* (*Clostridioides*) *difficile*, molecular epidemiology, bacterial spore, wastewater, sewage, biosolids, One Health

## Abstract

There is growing evidence that shows *Clostridium* (*Clostridioides*) *difficile* is a pathogen of One Health importance with a complex dissemination pathway involving animals, humans, and the environment. Thus, environmental discharge and agricultural recycling of human and animal waste have been suspected as factors behind the dissemination of Clostridium difficile in the community. Here, the presence of C. difficile in 12 wastewater treatment plants (WWTPs) in Western Australia was investigated. Overall, C. difficile was found in 90.5% (114/126) of raw sewage influent, 48.1% (50/104) of treated effluent, 40% (2/5) of reclaimed irrigation water, 100% (38/38) of untreated biosolids, 95.2% (20/21) of anaerobically digested biosolids, and 72.7% (8/11) of lime-amended biosolids. Over half of the isolates (55.3% [157/284]) were toxigenic, and 97 C. difficile ribotypes (RTs) were identified, with RT014/020 the most common (14.8% [42/284]). Thirteen C. difficile isolates with the toxin gene profile A^+^ B^+^ CDT^+^ (positive for genes coding for toxins A and B and the binary *C. difficile* transferase toxin [CDT]) were found, including the hypervirulent RT078 strain. Resistance to the antimicrobials fidaxomicin, vancomycin, metronidazole, rifaximin, amoxicillin-clavulanate, meropenem, and moxifloxacin was uncommon; however, resistance to clindamycin, erythromycin, and tetracycline was relatively frequent at 56.7% (161/284), 14.4% (41/284), and 13.7% (39/284), respectively. This study revealed that toxigenic C. difficile was commonly encountered in WWTPs and being released into the environment. This raises concern about the possible spillover of C. difficile into animal and/or human populations via land receiving the treated waste. In Western Australia, stringent measures are in place to mitigate the health and environmental risk of recycling human waste; however, further studies are needed to elucidate the public health significance of C. difficile surviving the treatment processes at WWTPs.

**IMPORTANCE** Clostridium difficile infection (CDI) is a leading cause of antimicrobial-associated diarrhea in health care facilities. Extended hospital stays and recurrences increase the cost of treatment and morbidity and mortality. Community-associated CDI (CA-CDI) cases, with no history of antimicrobial use or exposure to health care settings, are increasing. The isolation of clinically important C. difficile strains from animals, rivers, soil, meat, vegetables, compost, treated wastewater, and biosolids has been reported. The objective of this study was to characterize C. difficile in wastewater treatment plants (WWTPs) in Australia. We found that C. difficile can survive the treatment processes of WWTPs, and toxigenic C. difficile was being released into the environment, becoming a potential source/reservoir for CA-CDI.

## INTRODUCTION

*Clostridium* (*Clostridioides*) *difficile* is an anaerobic Gram-positive spore-forming bacillus that causes disease ranging from uncomplicated diarrhea to life-threatening pseudomembranous colitis, bowel perforation, sepsis, and death (1). Clostridium difficile is ranked as one of the most urgent antimicrobial resistance threats to public health by the U.S. Centers for Disease Control and Prevention (2, 3). Each year, C. difficile causes up to 500,000 infections and 29,000 deaths (~6% mortality rate) in the United States (4). In Australia, C. difficile infection (CDI) results in ~8,500 cases and 500 deaths per year (5, 6) and is estimated to cost the health care system an average of AUD (Australian dollars) $12,704 per hospitalization and a total of AUD $108 million per year (7).

The virulence of C. difficile is primarily attributed to toxins A and B, the genes for which are located on a pathogenicity locus (PaLoc) (8). An additional binary toxin (C. difficile transferase [CDT]), encoded on the CDT locus (CdtLoc) but not found in all strains, was reported to be associated with severe disease (9). Traditionally, CDI has been considered a hospital-associated (HA) infection related to old age and antimicrobial exposure, especially to those antimicrobials with activity against the commensal gut flora which normally protects against overgrowth of C. difficile by inhibiting spore germination, vegetative growth, and toxin production (1). Over the past 2 decades, the incidence of CDI has increased globally, with rates remaining high in many high-income countries with growing reports of outbreaks (1, 10). Previously rare community-associated CDI CA-CDI, defined as cases with symptom onset in the community and no history of hospitalization in the past 12 weeks or symptom onset within ≤48 h of hospital admission (11), has emerged to represent a sizable proportion of CDI cases (1). In the United States, CA-CDI accounts for 41% of all CDI cases, an increase of 4-fold from 1991 (12). In Australia, nearly 30% of all cases are CA, a 4-fold increase from 1995 to 2011 (5, 13).

Using whole-genome sequencing (WGS), a study in the United Kingdom revealed that 45% of C. difficile strains isolated from 957 CDI cases were genetically distinct from all previous isolates (14). This refuted the traditional notion that CDI was primarily a HA infection and suggested that the transmission of C. difficile involved sources/reservoirs outside the health care system. These are likely to be community based, such as foodborne and/or environmental acquisition, as clinically important C. difficile strains have been isolated from animals, meat, vegetables, compost, gardens, lawns, rivers, and lakes (15–17). This is in agreement with genomic studies in Europe and Australia that show (i) long-distance or cross-continental clonal transmission of C. difficile between humans and animals with ≤2 single nucleotide polymorphism (SNP) differences in their core genome (18, 19) and (ii) genetically closely related C. difficile strains isolated from humans, food, and the environment (retail potatoes, ready-to-eat salads, meat, compost, rivers, lakes, lawns, and the end products of wastewater treatment plants) (20–22). Taken together, this suggests a complex dissemination pathway of C. difficile between animals, humans, and the environment. Thus, the environmental discharge and agricultural recycling of human and animal waste were hypothesized to be factors behind the widespread dissemination of C. difficile in the community, which could lead to a rise in CDI.

Human sewage is treated through a series of physical, biological, and chemical processes at wastewater treatment plants (WWTPs) to reduce organic matter and pathogen loads. However, the robustness of spore-forming pathogens such as clostridia enables them to survive the treatment process with the potential to even flourish in the anaerobic digestive tanks commonly used to treat sludge (solid waste) from WWTPs. To date, few studies have investigated the presence and survival of C. difficile through WWTPs (23–26). The isolation of C. difficile strains in effluents (treated wastewater) has been described in the Czech Republic, Switzerland, Slovenia, England, and New Zealand (23–27). In Canada, Xu et al. isolated C. difficile from effluents and biosolids as well as sediments taken from rivers connected to the effluent discharge pipes (28). The occurrence of C. difficile in WWTPs in Australia has not been explored. Thus, the objective of this study was to isolate and characterize C. difficile from influent (untreated wastewater), effluent, reclaimed irrigation water, and biosolids from WWTPs in Western Australia (WA).

## RESULTS

### C. difficile prevalence.

Overall C. difficile recovery was 90.5% (114/126) from influent, 48.1% (50/104) from effluent, 40% (2/5) from reclaimed irrigation water, and 94.3% (66/70) from biosolids (100% [38/38] from untreated biosolids, 95.2% [20/21] from anaerobically digested biosolids, and 72.7% [8/11] from lime-amended biosolids) (Table 1). Proportions of C. difficile recovered from influent collected at the 12 different WWTPs were not significantly different between sites (*P* = 0.78; range, 75% to 100%), but differences were detected in effluent (*P* = 0.004; range, 0% to 100%) and biosolids (*P* = 0.01; range, 75.7% to 100%) from different WWTPs.

**TABLE 1:** Characteristics of wastewater treatment plants and the prevalence of C. difficile

WWTP^*a*^	Treatment process	Final effluent receiving body	Final biosolids application	Prevalence, % (*n*)			
				Influent	Effluent	Irrigation	Biosolids
W1	Preliminary, primary, secondary	Ocean		100 (11/11)	54.5 (6/11)		
W2	Preliminary, primary, secondary, anaerobic digestion of biosolids	Ocean, groundwater	Agricultural land	100 (11/11)	75.0 (3/4)		90.0 (9/10)^*b*^
W3	Preliminary, primary, secondary	Woodlot/wetland		87.5 (7/8)	30.0 (3/10)		
W4	Preliminary, primary, secondary	Groundwater		90.9 (10/11)	45.5 (5/11)		
W5	Preliminary, primary, secondary	Ocean, W7	Agricultural land	81.8 (9/11)	18.2 (2/11)		100 (14/14)^*c*^
W6	Preliminary, primary, secondary	Groundwater		100 (10/10)	81.8 (9/11)		
W7	Microfiltration, reverse osmosis membrane	Ocean, groundwater	Agricultural land	75.0 (9/12)	0.0 (0/4)		100 (12/12)^*c*^
W8	Preliminary, primary, secondary	Groundwater	Agricultural land	90.0 (9/10)	60.0 (6/10)		100 (12/12)^*c*^
W9	Preliminary, primary, secondary, filtration, chlorination, fluoridation, ultraviolet disinfection	Sport grounds, creek		90.9 (10/11)	10.0 (1/10)		
W10	Preliminary, primary	Ocean		90.0 (9/10)	100 (4/4)		
W11	Preliminary, primary, secondary, chlorination, lime amendment of biosolids	Ocean, sport grounds	Agricultural land	90.0 (9/10)	66.7 (6/9)	40.0 (2/5)	72.7 (8/11)^*d*^
W12	Preliminary, primary, secondary, anaerobic digestion of biosolids	Ocean, W7	Agricultural land	90.9 (10/11)	55.6 (5/9)		100 (11/11)^*b*^
** **							
Total				90.5 (114/126)	48.1 (50/104)	40.0 (2/5)	94.3 (66/70)

a WWTP, wastewater treatment plant.

b Anaerobically digested biosolids.

c Untreated biosolids.

d Lime-amended biosolids.

### Toxin gene profiling and ribotyping of C. difficile isolates.

Of the 284 C. difficile isolates recovered, 52.8% (150/284) contained *tcdA* and *tcdB* genes coding for toxins A and B (A^+^ B^+^), of which 8.7% (13/150) were also positive for binary toxin genes (*cdtA* and *cdtB*) (CDT^+^). A total of 127 isolates (44.7%) were nontoxigenic (A^−^ B^−^ CDT^−^). The remaining seven isolates yielded the following toxin gene profiles: A^−^ B^−^ CDT^+^ (1.4% [4/284]) and A^−^ B^+^ CDT^+^ (1.1% [3/284]). Toxigenic strains were isolated from 47.6% (60/126) of influent, 30.8% (32/104) of effluent, 71.4% (50/70) of biosolids, and none of the five irrigation water samples.

**FIG 1:**
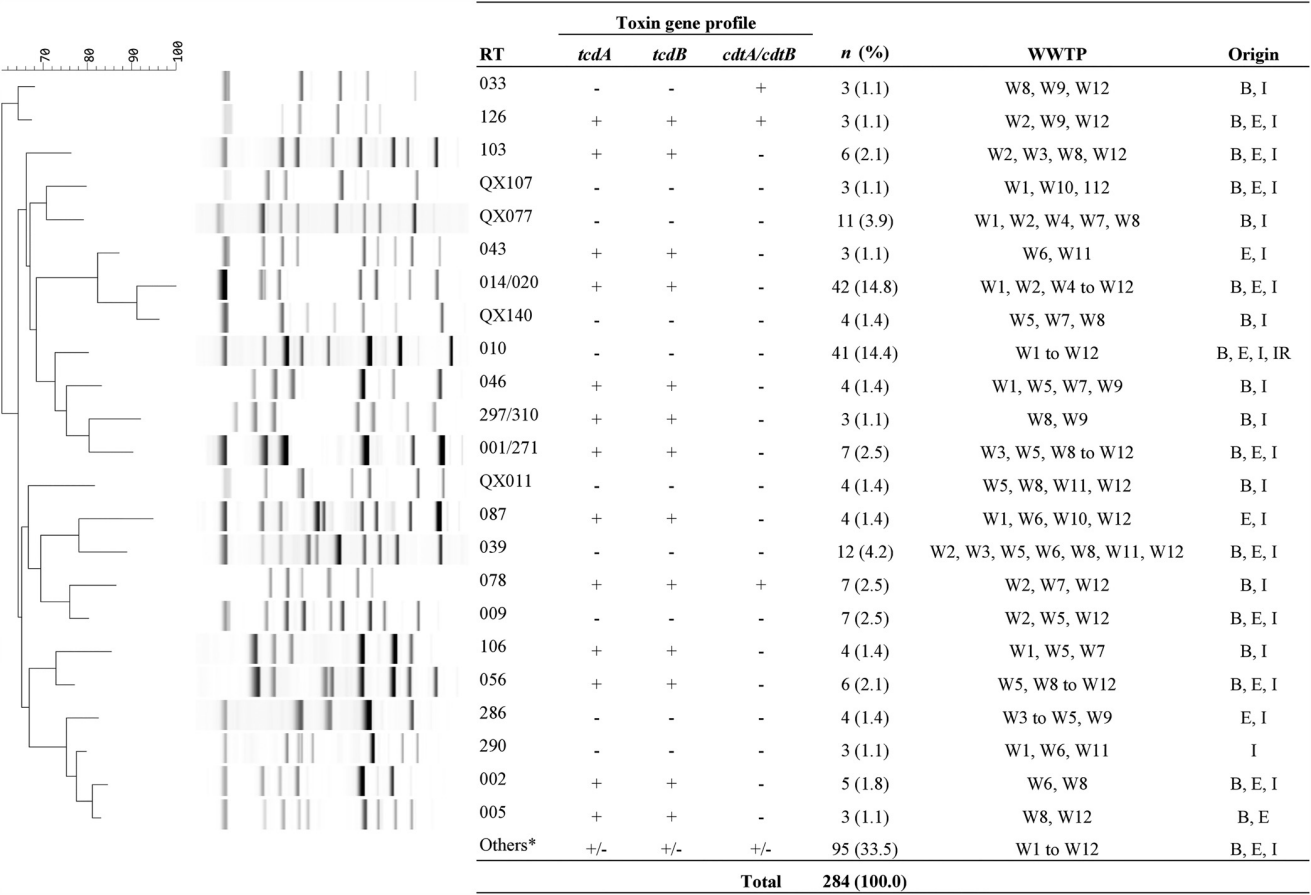
Summary of C. difficile ribotyping patterns and toxin gene profiles of isolates obtained from wastewater treatment plants in WA. Ribotyping pattern analysis was performed with a neighbor-joining tree using Pearson correlation. WWTP, wastewater treatment plant; B, biosolids; E, effluents; I, influents; IR, reclaimed irrigation water. *, “Others” represents RTs with a frequency of ≤2. These were RT no. 003, 012, 015/193, 018, 026/118, 051, 053, 064, 070, 081, 101, 125, 127, 137, 247, 281, 584, and 605 and local RTs (including 17 singletons and QX no. 002, 024, 025, 026, 029, 042, 051, 057, 068, 076, 086, 087, 099, 121, 141, 161, 189, 195, 210, 211, 229, 243, 365, 372, 395, 488, 491, 509, 532, 541, 605, 610, 629, 656, 657, 658, 659, and 660).

Ninety-seven C. difficile ribotypes (RTs) were identified, 37 (38.1%) of which were internationally recognized, 43 (44.3%) were classified with internal nomenclature and 17 (17.5%) were novel (Fig. 1). The majority of the novel strains (64.7% [11/17]) were nontoxigenic. The most common RT found was RT014/020 (A^+^ B^+^ CDT^−^), which comprised 14.8% (42/284) of the isolates, followed by RT010 (A^−^ B^−^ CDT^−^) (14.4% [41/284]). The 13 C. difficile strains with toxin profile A^+^ B^+^ CDT^+^ were RT078 (*n *=* *7), RT126 (*n* = 3), RT127 (*n* = 2), and QX 656 (*n* = 1). Toxigenic C. difficile RT014/020 represented the majority of isolates found in influent (12.5% [16/128]), effluent (25.5% [13/51]), and biosolids (12.6% [13/103]) but not in irrigation water (0% [0/2]).

### Antimicrobial susceptibility of C. difficile isolates.

Most isolates were susceptible to the antimicrobials recommended for treatment of CDI (Table 2). Vancomycin and metronidazole inhibited 98.2% and 100% of the isolates, respectively. The *in vitro* activity of rifaximin was the most potent (MIC_50_/MIC_90_, 0.03/0.03 mg/L), followed by fidaxomicin (MIC_50_/MIC_90_, 0.25/0.25 mg/L), metronidazole (MIC_50_/MIC_90_, 0.25/0.5 mg/L), and vancomycin (MIC_50_/MIC_90_, 2/2 mg/L). All isolates were susceptible to fidaxomicin, metronidazole, and amoxicillin-clavulanic acid. The proportions of isolates resistant to vancomycin (1.8% [5/284]), rifaximin (1.4% [4/284]), meropenem (0.35% [1/284]), and moxifloxacin (2.5% [7/284]) were low. Forty-one isolates (14.4%) were resistant to erythromycin, all with a MIC of >256 mg/L. These isolates were RT039 (*n *=* *6), RT014/020 (*n* = 4), QX011 (*n* = 4), RT046 (*n* = 3), RT009 (*n* = 2), RT078 (*n* = 2), RT126 (*n* = 2), RT247 (*n* = 2), QX024 (*n* = 2), QX395 (*n* = 2), RT001/271 (*n* = 1), RT005 (*n* = 1), RT010 (*n* = 1), RT051 (*n* = 1), RT053 (*n* = 1), RT056 (*n* = 1), RT127 (*n* = 1), QX099 (*n* = 1), QX140 (*n* = 1), QX658 (*n* = 1), and two novel RTs. Resistance to tetracycline was observed in 13.7% (39/284) of the isolates, including RT078 (*n *=* *6), RT014/020 (*n* = 4), RT039 (*n* = 4), RT046 (*n* = 3), QX011 (*n* = 3), RT010 (*n* = 2), RT126 (*n* = 2), RT247 (*n* = 2), QX395 (*n* = 2), RT005 (*n* = 1), RT009 (*n* = 1), RT012 (*n* = 1), RT106 (*n* = 1), RT127 (*n* = 1), QX024 (*n* = 1), QX107 (*n* = 1), QX141 (*n* = 1), QX658 (*n* = 1), and two novel strains. Over half the isolates (56.7% [161/284]) were resistant to clindamycin. Among the frequently isolated RTs, resistance to clindamycin was most common in the nontoxigenic isolate RT039 (91.7% [11/12]), with a geometric mean (GM) of 27.4 mg/L compared to 6.75 mg/L of all isolates. C. difficile RT039, a nontoxigenic strain, also displayed greater resistance to tetracycline (GM of 3.36 mg/L versus 0.56 mg/L for all isolates).

**TABLE 2:** Antimicrobial susceptibility data for C. difficile isolates by ribotype^*a*^

RT	Antimicrobial	MIC (mg/L)		GM	Breakpoint (mg/L)			Isolate resistance					
								S		I		R	
		Range	MIC_50_/MIC_90_		S	I	R	%	*n*	%	*n*	%	*n*
All (*n* = 284)	FDX	0.03 to 0.5	0.25/0.25	0.16			≥1	100.0	284				
	VAN	1 to 4	2/2	1.45	≤2		>2	98.2	279			1.8	5
	MTZ	0.125 to 1	0.25/0.5	0.26	≤2		>2	100.0	284				
	RFX	0.015 to >64	0.03/0.03	0.03			≥32	98.6	280			1.4	4
	CLI	0.125 to >256	8/32	6.75	≤2	4	≥8	12.3	35	31.0	88	56.7	161
	ERY	0.25 to >256	2/>256	1.51			>8	85.6	243			14.4	41
	AMC	0.125 to 2	0.5/1	0.61	≤4	8	≥16	100.0	284				
	MEM	1 to 16	2/4	2.34	≤4	8	≥16	98.6	280	1.1	3	0.35	1
	MXF	1 to 32	2/2	2.06	≤2	4	≥8	94.7	269	2.8	8	2.5	7
	TET	0.125 to 64	0.25/16	0.56	≤4	8	≥16	83.8	238	2.5	7	13.7	39

RT014/020 (*n* = 42)	FDX	0.03 to 0.5	0.25/0.25	1.70			≥1	100.0	42				
	VAN	1 to 2	1/2	1.37	≤2		>2	100.0	42				
	MTZ	0.125 to 0.5	0.25/0.25	0.25	≤2		>2	100.0	42				
	RFX	0.015 to 16	0.03/0.03	0.03			≥32	100.0	42				
	CLI	0.5 to >256	8/16	6.61	≤2	4	≥8	9.5	4	23.8	10	66.7	28
	ERY	0.25 to >256	2/4	1.58			>8	90.5	38			9.5	4
	AMC	0.25 to 1	0.5/0.5	0.51	≤4	8	≥16	100.0	42				
	MEM	1 to 4	2/2	2.07	≤4	8	≥16	100.0	42				
	MXF	2 to 2	2/2	2.00	≤2	4	≥8	100.0	42				
	TET	0.125 to 64	0.25/1	0.42	≤4	8	≥16	90.5	38			9.5	4

RT010 (*n* = 41)	FDX	0.03 to 0.5	0.25/0.5	0.21			≥1	100.0	41				
	VAN	1 to 2	1/2	1.31	≤2		>2	100.0	41				
	MTZ	0.25 to 0.5	0.25/0.25	0.26	≤2		>2	100.0	41				
	RFX	0.015 to 0.03	0.03/0.03	0.03			≥32	100.0	41				
	CLI	1 to 64	8/16	6.42	≤2	4	≥8	9.8	4	36.6	15	53.7	22
	ERY	1 to >256	2/2	1.74			>8	97.6	40			2.4	1
	AMC	0.5 to 1	1/1	0.95	≤4	8	≥16	100.0	41				
	MEM	2 to 4	2/4	2.76	≤4	8	≥16	100.0	41				
	MXF	2 to 4	2/2	2.07	≤2	4	≥8	95.1	39	4.9	2		
	TET	0.125 to 32	0.25/0.5	0.31	≤4	8	≥16	95.1	39			4.9	2

RT039 (*n* = 12)	FDX	0.125 to 0.5	0.25/0.25	0.22			≥1	100.0	12				
	VAN	1 to 2	1/2	1.33	≤2		>2	100.0	12				
	MTZ	0.25 to 0.5	0.5/0.5	0.42	≤2		>2	100.0	12				
	RFX	0.015 to 0.03	0.03/0.03	0.03			≥32	100.0	12				
	CLI	4 to >256	16/>256	27.43	≤2	4	≥8			8.3	1	91.7	11
	ERY	2 to >256	2/>256	2.00			>8	50.0	6			50.0	6
	AMC	0.5 to 1	1/1	0.94	≤4	8	≥16	100.0	12				
	MEM	2 to 4	2/4	2.67	≤4	8	≥16	100.0	12				
	MXF	2 to 2	2/2	2.00	≤2	4	≥8	100.0	12				
	TET	0.25 to 16	2/16	3.36	≤4	8	≥16	66.7	8			33.3	4

88 “other” RTs (*n* = 189)	FDX	0.03 to 0.5	0.25/0.25	0.15			≥1	100.0	189				
	VAN	1 to 4	2/2	1.51	≤2		>2	97.4	184			2.6	5
	MTZ	0.125 to 1	0.25/0.5	0.25	≤2		>2	100.0	189				
	RFX	0.015 to >64	0.03/0.03	0.03			≥32	97.9	185			2.1	4
	CLI	0.125 to >256	8/32	6.40	≤2	4	≥8	14.3	27	32.8	62	52.9	100
	ERY	0.25 to >256	2/>256	1.43			>8	84.1	159			15.9	30
	AMC	0.125 to 2	0.5/1	0.57	≤4	8	≥16	100.0	189				
	MEM	1 to 16	2/4	2.31	≤4	8	≥16	97.9	185	1.6	3	0.5	1
	MXF	1 to 32	2/2	2.07	≤2	4	≥8	93.1	176	3.2	6	3.7	7
	TET	0.125 to 64	0.25/16	0.61	≤4	8	≥16	81.0	153	3.7	7	15.3	29

a RT, ribotype; FDX, fidaxomicin; VAN, vancomycin; MTZ, metronidazole; RFX, rifaximin; CLI, clindamycin; ERY, erythromycin; AMC, amoxicillin-clavulanic acid; MEM, meropenem; MXF, moxifloxacin; TET, tetracycline; GM, geometric mean; S, susceptible; I, intermediate; R, resistant.

Multidrug-resistance (MDR), defined as resistance to ≥3 antimicrobial classes, was observed among 7.0% (20/284) of C. difficile isolates. These were RT046 (*n *=* *3), QX011 (*n* = 3), RT014/020 (*n* = 2), RT078 (*n* = 2), RT005 (*n* = 1), RT039 (*n* = 1), RT051 (*n* = 1), RT126 (*n* = 1), RT127 (*n* = 1), RT247 (*n* = 1), QX024 (*n* = 1), QX395 (*n* = 1), QX658 (*n* = 1), and one novel strain. The most common MDR profiles were resistance to clindamycin, erythromycin, and tetracycline (85.0% [17/20]). Two isolates, RT005 and QX011, were resistant to five antimicrobials (rifaximin, clindamycin, erythromycin, moxifloxacin, and tetracycline). Two isolates (RT126 and QX011) were resistant to clindamycin, erythromycin, moxifloxacin, and tetracycline; and a single RT046 strain was resistant to rifaximin, clindamycin, erythromycin and tetracycline.

## DISCUSSION

In this study, we found that toxigenic C. difficile was commonly encountered in WWTPs and was being released into the environment, consistent with earlier European studies. In Switzerland, Romano et al. isolated C. difficile in 100% of their influent and treated effluent samples from nine WWTPs, with 85% of the strains being toxigenic (26). This raised concerns about the possible contamination of local rivers that were receiving the treated effluents and the safety of reusing treated effluents. In 2015, Steyer et al. performed a 1-year survey on the occurrence of enteric pathogens in effluent from a conventional two-stage activated sludge WWTP in Slovenia (23). Astroviruses were not found in August and September, and hepatitis A and E viruses were not detected at all, while rotaviruses, noroviruses, sapoviruses, and C. difficile were detected in all samples collected throughout the study period. In total, 121 C. difficile strains with 32 different RTs were isolated in the study, of which RT014/020 and RT010 were the most prevalent. This confirmed the authors’ previous findings of C. difficile being widely distributed in Slovenian rivers, including RT014, with a positive correlation with increased population densities (29). Thus, the release of effluent into local rivers was suspected of being a potential source of C. difficile contamination. In 2018, 186 C. difficile strains isolated from effluent samples from 18 WWTPs in England were sequenced and compared with 70 clinical isolates using phylogenetic analysis (24). C. difficile isolates from human clinical cases and WWTPs were genetically highly related. Overall, these studies confirmed the extensive release of toxigenic C. difficile into surface waters and question the need to improve strategies for the removal of bacterial spores associated with causing human diseases from the effluent before release into the environment.

In comparison, our study yielded a similarly high prevalence of C. difficile in influent (90.5%) but the overall isolation of C. difficile in effluent was lower at 48.1%. This was likely due to the inclusion of WWTPs that performed tertiary (e.g., sand filtration and chlorination) and advanced water (e.g., membrane filtration and reverse osmosis) treatment. All effluent samples from W7, a WWTP that uses membrane filtration and reverse osmosis to produce high-quality reclaimed water, did not contain C. difficile, and only 10% of effluent from W9 had C. difficile, likely attributed to the plant performing membrane filtration, chlorination, and ultraviolet disinfection in addition to the traditional activated sludge treatment process. Effluents from the remaining WWTPs had a C. difficile prevalence ranging between 18.2% and 100%, averaging 58.7%. However, this finding should be interpreted with some care given the small number of samples collected from each WWTP. While the release of effluent into water bodies can lead to contamination and therefore potentially contribute to CA-CDI in at-risk populations upon exposure to environmental C. difficile spores, we suspect the risk of acquiring CDI via effluent discharged into the ocean is low in WA as, in our recent study, C. difficile was not found in any of the 89 seawater samples collected along the coast of WA near Perth, the state capital (30). Furthermore, the use of effluent as irrigation water is not widespread in WA, with only a small number of local councils using it to irrigate their sport grounds and public spaces. However, a notable proportion of our effluent was used to replenish the groundwater, which is commonly extracted to irrigate golf courses, public spaces, and residential lawns. The presence and bacterial load of C. difficile in groundwater, as well as fields that use groundwater as irrigation, were not studied here and hence require further investigation.

In Canada, Xu et al. reported a high prevalence of C. difficile in raw sludge (92%), anaerobically digested sludge (96%), lime-amended biosolids (73%), and sediments (39%) from rivers that received the effluent that has been chlorinated, passed through sand filters, and dechlorinated prior to disposal into rivers (28). This is comparable with our findings of C. difficile in 100% of untreated biosolids, 95.2% of anaerobically digested biosolids, and 72.7% of lime-amended biosolids. In Australia, the management of biosolids is strictly regulated, with pathogen (P1 to P4) and chemical contaminant (C1 to C3) grading determining their suitability for different end uses (31). The highest-quality biosolids (P1C1) have no restrictions in application, and biosolids with a P1C2 grading can be used in land application for crops that may be consumed raw. Midquality biosolids (P2C2 and P3C2) can be used for landscaping, horticulture, forestry and pasture, and crops that are processed before consumption and have no direct contact with soil. Low-quality biosolids with a P4C3 grading are destined for composting or landfill. The application of biosolids with such a high prevalence of C. difficile may lead to widespread dissemination of C. difficile in the environment. However, none of the biosolids tested in this study was of the highest quality (P1C1) with unrestricted use as the biosolids produced by the WWTPs in WA were predominantly midquality with a P2C2 or P3C2 grade. For biosolids to be graded as P2 or P3, they must undergo treatment such as (i) composting according to the Australian Standard AS 4454, which is reaching an internal temperature of 55°C for 3 consecutive days with a minimum of three turns for lower-risk materials such as plant and vegetation, and 15 days with a minimum of five turns for high-risk materials, or (ii) the addition of lime so that the pH is maintained at >12 for 72 h. In addition, the effectiveness of these treatments at reducing pathogen levels must be validated by testing the level of fecal indicator organisms (Escherichia coli and viable helminths) (31). In this study, although the level of C. difficile was not known, the survival of C. difficile in biosolids treated with lime was seen with a 72.7% prevalence. In addition, composting is insufficient at reducing C. difficile spores to an undetectable level (32, 33). This was attributed to C. difficile being a sporeformer capable of withstanding high temperatures and harsh environmental conditions (33). In fact, C. difficile has been isolated in commercially composted products in Australia and the United States at a prevalence of 22.5% and 35.9%, respectively (34, 35). In WA, a conservative approach was adopted to protect the environment from nutrient runoff and reduce the risk of exposure to the general public. Currently, biosolids are no longer applied to forestry or pasture, and they are only supplied to agriculture land where crops will be processed before consumption and have no direct contact with the soil. Thus, risk of exposure to C. difficile via biosolids is presumably low. Nevertheless, the dissemination of C. difficile via commercial compost is likely and worth investigating. The length of time that C. difficile spores can persist on pasture and in biosolid-incorporated soil also remains to be determined.

In agreement with Moradigaravand et al., we found an overlap between C. difficile genotypes isolated from WWTPs and those isolated from humans in the same area, with 65 out of 97 RTs found in WWTPs also isolated from hospital patients (24). Having been isolated from influent, effluent, and biosolids across 11 WWTPs, C. difficile RT014/020 was the most common among all strains (14.8%). C. difficile RT014 is a lineage of One Health importance, well established in humans, pigs, and the environment in Australia (19). Nationwide, C. difficile RT014/020 accounts for ~30% of all CDI cases in humans and 23% of isolates from neonatal pigs (36, 37). In WA, C. difficile RT014/020 also represents a significant proportion of C. difficile isolates from lawn (39%), compost (10%), hospital gardens (14%), home gardens (21%), root vegetables (7%), and water bodies (11%) (30, 34, 38–41). Although the zoonotic and environmental transmission of C. difficile is still being debated, recent data suggest long-distance transmission of C. difficile between animals, environmental reservoirs, and humans (18, 19, 30, 34, 42, 43). In our recent study where 142 C. difficile RT014 strains from humans, animals, compost, hospital gardens, lawns, root vegetables, shoes, and water bodies were sequenced and analyzed, extensive coclustering of human, animal and environmental strains was found, with 9% of the human strains having a clonal relationship (≤2 SNPs) indicative of direct transmission and 60% closely related (≤9 SNPs) to at least one animal or environmental strain (22). We suspect the land application of biosolids and animal manure has aided in the dissemination of C. difficile in the environment and may have contributed to a rise in CA-CDI. In addition, it is worth noting that C. difficile RT078 isolated from the biosolids of W2, W7, and W12 is a hypervirulent strain associated with severe CDI in a younger population and more frequently CA-CDI in Europe (44). It was the third most common RT of C. difficile isolated from CDI patients in 34 European countries (45). Even though human CDI cases caused by RT078 are uncommon in Australia (36), the finding of RT078 in biosolids is a particular concern from a public health standpoint.

Not surprisingly, the antimicrobial resistance (AMR) profile of C. difficile from WWTPs in Australia was similar to those from humans, with no or low-level resistance to fidaxomicin, vancomycin, metronidazole, rifaximin, amoxicillin-clavulanate, meropenem, and moxifloxacin but high-level resistance to clindamycin (46, 47). Interestingly, the nontoxigenic C. difficile RT039 strain displayed a high level of resistance to clindamycin and tetracycline. Due to RT039 being nontoxigenic, it is usually not reported from human clinical samples (46, 47); however, it is a relatively common RT found in the environment in Australia, although greater resistance to any antimicrobial agents has never been reported (48). Nontoxigenic C. difficile strains are highly prevalent in Asia, and many of these strains are resistant to multiple antimicrobials, possibly due to inappropriate antimicrobial use in the region (49). These C. difficile RT039 strains may be of Asian origin and could have either acquired AMR genes via horizontal gene transfer from other microbes in the host intestine or from the consortium of pathogenic/commensal microbes in WWTPs. This could include mobile genetic elements such as transposons Tn*6194* (harboring *ermB*) and Tn*6190* (harboring *tetM*) which are known to transfer resistance to clindamycin and tetracycline, respectively (19, 42). The presence of diverse selective pressures in a WWTP could create favorable conditions for the transfer of AMR genes and the proliferation of AMR bacteria. AMR is a growing global health threat, yet environmental surveillance of AMR is largely lacking and WWTPs could potentially be a focal point in the fight against AMR.

Although a low number of samples per sample type (influent, effluent, biosolids, and reclaimed water) was collected from each of the WWTPs and quantification of C. difficile was not performed, the importance of effluent and biosolids in C. difficile release into the environment was clearly demonstrated in our study. In summary, we showed that WWTP influent, effluent, and biosolids in WA contained toxigenic C. difficile strains belonging to RTs found in human CDI cases. Thus, the release of effluent and biosolids can create a source or reservoir of community infection. Future studies are needed to determine the fate of C. difficile traversing the treatment process and the public health significance of C. difficile surviving the treatment processes at WWTPs and commercial composting processes. With climate change and increased water scarcity, the development of efficient wastewater treatment and recycling practices is imperative to securing a sustainable future. WWTPs should have a central role in environmental surveillance of AMR and emerging infectious diseases, and this will require a multidisciplinary One Health approach.

## MATERIALS AND METHODS

### Setting.

In Australia, the processing of wastewater follows a specific sequence according to sewerage system guidelines (50). In WA, 80% of the wastewater collected is treated in the following ways: raw influent arriving at the WWTP goes through a preliminary treatment process that involves filtering the wastewater through screens and grit tanks to remove any large inorganic objects. The wastewater then flows into the primary sedimentation tank (also known as the settling tank or clarifier), where particles in the water gradually sink to the bottom of the tank to form sludge. The wastewater then flows into the aeration tank, where microbes feed on oxygen, organic matter, and any dissolved nutrients to produce carbon dioxide, nitrogen, and more sludge. It then flows into the secondary sedimentation tank, which is the final stage of treatment in most treatment plants before the treated effluent is released into the environment. In WA, the effluent is either returned to the ocean via large offshore pipes with small holes to ensure the effluent is evenly dispersed into the sea, used to replenish the groundwater, or undergoes further advanced treatment before being recycled as irrigation water for sports grounds, public open spaces, and nonfood crops (e.g., trees, turf, and flowers), flushing the toilet, washing clothes, maintaining wetlands, and industrial reuse of high-quality reclaimed water, such as for use in the oil refining sector. The use of recycled water follows guidelines from the WA Department of Health (51). The sludge from WWTPs is either (i) collected and transferred to an anaerobic digester, where organic matter is broken down into biogas and digested biosolids, or (ii) dewatered using centrifugation with or without the addition of lime to reach a pH of >12. The management and subsequent use of biosolids follow guidelines from the WA Department of Water and Environment Regulation (previously known as the Department of Environment and Conservation) (31). These guidelines include a series of measures designed to manage the health and environmental risks associated with recycling biosolids, including determining the soil types, depth to groundwater, proximity to sensitive land and water resources, buffer distances to neighboring protected areas, timing/seasons of application, and slope of the land to prevent runoff (31).

### Sampling.

Wastewater treatment plant samples in WA are routinely tested for the presence of selected microbes and heavy metals, and this study conveniently used their excess samples. Samples from the same WWTP were collected on the same day, once every 2 weeks, between January 2020 and June 2020. A total of 126 influents, 104 effluents, 5 irrigation water samples, and 70 biosolids (38 untreated, 21 anaerobically digested, and 11 amended with lime) were collected from 12 WWTPs (W1 to W12) in WA (Table 1). Influent and effluent samples were collected from all 12 WWTPs. Biosolids from W5, W7, and W8 were untreated. Anaerobically digested biosolids were from W2 and W12. Lime-amended biosolids and reclaimed irrigation water were from W11.

### Culture conditions.

Ten milliliters of each influent, effluent, and reclaimed irrigation water sample was filtered through a 0.45-μm-pore cellulose membrane (Pall Corporate, product ID 4761) using a manifold filtration system. The membrane was then placed in 90 mL brain heart infusion broth supplemented with 5 g/L yeast extract, 1 g/L l-cysteine, 1 g/L taurocholic acid, 250 mg/L cycloserine, and 8 mg/L cefoxitin (BHIB-S) (PathWest Media, Mt Claremont, WA, Australia) and incubated anaerobically in a Don Whitley Scientific, Ltd. (Otley, Yorkshire, United Kingdom), A35 anaerobic chamber (10% hydrogen, 10% carbon dioxide, 80% nitrogen) at 35°C for 7 days. Approximately 5 g of biosolid sample was added to 90 mL BHIB-S and incubated anaerobically as described above. After incubation, 2 mL of broth was alcohol shocked by mixing an equal volume of absolute alcohol and standing for 1 h. The suspension was then centrifuged at 3,800 × *g* for 10 min, and a 10-μL loopful of pellet was plated onto C. difficile ChromID agar (bioMérieux, France). The plates were incubated anaerobically and examined at 48 h. Unless there were colonies with different morphologies, one presumptive C. difficile colony (small, irregular with a raised umbonate profile, colored or not) per ChromID plate was subcultured onto a horse blood agar plate for identification based on colony morphology, odor, and chartreuse fluorescence under UV light (~360 nm) (38).

### Toxin gene profiling and ribotyping.

Crude bacterial template DNA for toxin profiling was prepared by resuspension of cells in a 5% (wt/vol) solution of Chelex-100 resin (Sigma-Aldrich, Castle Hill, NSW, Australia). All isolates were screened by PCR for the presence of toxin A (*tcdA*), toxin B (*tcdB*), and binary toxin (*cdtA* and *cdtB*) genes (38). PCR ribotyping was performed as previously described (52). PCR ribotyping products were concentrated using a Qiagen MinElute PCR purification kit (Qiagen, Hilden, Germany). Visualisation of PCR products was performed with QIAxcel ScreenGel software (Qiagen, Hilden, Germany). Using the BioNumerics software package v.7.5 (Applied Maths, Sint-Martens-Latem, Belgium), the ribotyping patterns generated were compared to a reference library that consisted of over 16,000 C. difficile strains, including 54 internationally recognized RTs from the Anaerobe Reference Laboratory (ARL) (Cardiff, United Kingdom) and the European Centre for Disease Prevention and Control (ECDC) collection. Isolates that gave patterns that did not correspond to any internationally recognized RTs in our library but had previously been isolated by our laboratory were assigned an internal nomenclature prefixed with “QX.” RTs that were new to our library were assigned a new QX number.

### Antimicrobial susceptibility testing.

MICs of a panel of 10 antimicrobial agents were determined by an agar incorporation method as described by the Clinical and Laboratory Standards Institute (CLSI) guidelines (53, 54). The panel included fidaxomicin, vancomycin, metronidazole, rifaximin, clindamycin, erythromycin, amoxicillin-clavulanic acid, moxifloxacin, meropenem, and tetracycline. The clinical breakpoints for vancomycin and metronidazole were those recommended by the European Committee on Antimicrobial Susceptibility Testing (EUCAST) (55). The breakpoint for fidaxomicin of ≥1 mg/L was proposed by the European Medicines Agency (EMA) (56). Rifaximin resistance (≥32 mg/L) was described previously (57), and the breakpoints for clindamycin, erythromycin, amoxicillin-clavulanic acid, moxifloxacin, meropenem, and tetracycline were those provided by the CLSI (53).

### Statistical analysis.

Fisher’s exact test was used, where appropriate, to compare the prevalence of C. difficile in samples from different WWTPs.

## References

[1] Rupnik M, Wilcox MH, Gerding DN. 2009. *Clostridium difficile* infection: new developments in epidemiology and pathogenesis. Nat Rev Microbiol 7:526–536. doi:10.1038/nrmicro2164. 19528959

[2] Centers for Disease Control and Prevention. 2013. Antibiotic resistance threats in the United States, 2013. Department of Health and Human Services, Atlanta, GA.

[3] Centers for Disease Control and Prevention. 2019. Antibiotic resistance threats in the United States 2019. Department of Health and Human Services, Atlanta, GA.

[4] Lessa FC, Mu Y, Bamberg WM, Beldavs ZG, Dumyati GK, Dunn JR, Farley MM, Holzbauer SM, Meek JI, Phipps EC, Wilson LE, Winston LG, Cohen JA, Limbago BM, Fridkin SK, Gerding DN, McDonald LC. 2015. Burden of *Clostridium difficile* infection in the United States. N Engl J Med 372:825–834. doi:10.1056/NEJMoa1408913. 25714160 PMC10966662

[5] Slimings C, Armstrong P, Beckingham WD, Bull AL, Hall L, Kennedy KJ, Marquess J, McCann R, Menzies A, Mitchell BG, Richards MJ, Smollen PC, Tracey L, Wilkinson IJ, Wilson FL, Worth LJ, Riley TV. 2014. Increasing incidence of *Clostridium difficile* infection, Australia, 2011–2012. Med J Aust 200:272–276. doi:10.5694/mja13.11153. 24641152

[6] Australian Commission on Safety and Quality in Health Care. 2020. *Clostridioides difficile* infection: 2018 data snapshot. Australian Commission on Safety and Quality in Health Care, Sydney, Australia.

[7] Chen Y, Glass K, Liu B, Korda RJ, Riley TV, Kirk MD. 2017. Burden of *Clostridium difficile* infection: associated hospitalization in a cohort of middle-aged and older adults. Am J Infect Control 45:508–511. doi:10.1016/j.ajic.2016.12.006. 28089675

[8] Braun V, Hundsberger T, Leukel P, Sauerborn M, von Eichel-Streiber C. 1996. Definition of the single integration site of the pathogenicity locus in *Clostridium difficile*. Gene 181:29–38. doi:10.1016/s0378-1119(96)00398-8. 8973304

[9] Barbut F, Decre D, Lalande V, Burghoffer B, Noussair L, Gigandon A, Espinasse F, Raskine L, Robert J, Mangeol A, Branger C, Petit JC. 2005. Clinical features of *Clostridium difficile*-associated diarrhoea due to binary toxin (actin-specific ADP-ribosyltransferase)-producing strains. J Med Microbiol 54:181–185. doi:10.1099/jmm.0.45804-0. 15673514

[10] Collins DA, Sohn KM, Wu Y, Ouchi K, Ishii Y, Elliott B, Riley TV, Tateda K, *Clostridioides difficile* Asia-Pacific Study Group. 2020. *Clostridioides difficile* infection in the Asia-Pacific region. Emerg Microbes Infect 9:42–52. doi:10.1080/22221751.2019.1702480. 31873046 PMC6968625

[11] McDonald LC, Coignard B, Dubberke E, Song XY, Horan T, Kutty PK, Ad Hoc Clostridium difficile Surveillance Working Group. 2007. Recommendations for surveillance of *Clostridium difficile*-associated disease. Infect Control Hosp Epidemiol 28:140–145. doi:10.1086/511798. 17265394

[12] Khanna S, Pardi DS, Aronson SL, Kammer PP, Orenstein R, St Sauver JL, Harmsen WS, Zinsmeister AR. 2012. The epidemiology of community-acquired *Clostridium difficile* infection: a population-based study. Am J Gastroenterol 107:89–95. doi:10.1038/ajg.2011.398. 22108454 PMC3273904

[13] Riley TV, Cooper M, Bell B, Golledge CL. 1995. Community-acquired *Clostridium difficile*-associated diarrhea. Clin Infect Dis 20:S263–S265. doi:10.1093/clinids/20.Supplement_2.S263. 7548570

[14] Eyre DW, Cule ML, Wilson DJ, Griffiths D, Vaughan A, O'Connor L, Ip CLC, Golubchik T, Batty EM, Finney JM, Wyllie DH, Didelot X, Piazza P, Bowden R, Dingle KE, Harding RM, Crook DW, Wilcox MH, Peto TEA, Walker AS. 2013. Diverse sources of *C. difficile* infection identified on whole-genome sequencing. N Engl J Med 369:1195–1205. doi:10.1056/NEJMoa1216064. 24066741 PMC3868928

[15] Weese JS. 2020. *Clostridium* (*Clostridioides*) *difficile* in animals. J Vet Diagn Invest 32:213–221. doi:10.1177/1040638719899081. 31904312 PMC7081495

[16] Rodriguez-Palacios A, Mo KQ, Shah BU, Msuya J, Bijedic N, Deshpande A, Ilic S. 2020. Global and historical distribution of *Clostridioides difficile* in the human diet (1981–2019): systematic review and meta-analysis of 21886 samples reveal sources of heterogeneity, high-risk foods, and unexpected higher prevalence toward the tropic. Front Med (Lausanne) 7:9. doi:10.3389/fmed.2020.00009. 32175321 PMC7056907

[17] Warriner K, Xu C, Habash M, Sultan S, Weese SJ. 2017. Dissemination of *Clostridium difficile* in food and the environment: significant sources of *C. difficile* community-acquired infection? J Appl Microbiol 122:542–553. doi:10.1111/jam.13338. 27813268

[18] Knetsch CW, Kumar N, Forster SC, Connor TR, Browne HP, Harmanus C, Sanders IM, Harris SR, Turner L, Morris T, Perry M, Miyajima F, Roberts P, Pirmohamed M, Songer JG, Weese JS, Indra A, Corver J, Rupnik M, Wren BW, Riley TV, Kuijper EJ, Lawley TD. 2018. Zoonotic transfer of *Clostridium difficile* harboring antimicrobial resistance between farm animals and humans. J Clin Microbiol 56:e01384-17. doi:10.1128/JCM.01384-17. 29237792 PMC5824051

[19] Knight DR, Squire MM, Collins DA, Riley TV. 2016. Genome analysis of *Clostridium difficile* PCR ribotype 014 lineage in Australian pigs and humans reveals a diverse genetic repertoire and signatures of long-range interspecies transmission. Front Microbiol 7:2138. doi:10.3389/fmicb.2016.02138. 28123380 PMC5225093

[20] Rodriguez C, Taminiau B, Avesani V, Van Broeck J, Delmee M, Daube G. 2014. Multilocus sequence typing analysis and antibiotic resistance of *Clostridium difficile* strains isolated from retail meat and humans in Belgium. Food Microbiol 42:166–171. doi:10.1016/j.fm.2014.03.021. 24929733

[21] Romano V, Pasquale V, Lemee L, El Meouche I, Pestel-Caron M, Capuano F, Buono P, Dumontet S. 2018. *Clostridioides difficile* in the environment, food, animals and humans in southern Italy: occurrence and genetic relatedness. Comp Immunol Microbiol Infect Dis 59:41–46. doi:10.1016/j.cimid.2018.08.006. 30290886

[22] Lim S-C, Collins DA, Imwattana K, Knight DR, Perumalsamy S, Hain-Saunders NMR, Putsathit P, Speers D, Riley TV. 2022. Whole-genome sequencing links *Clostridium* (*Clostridioides*) *difficile* in a single hospital to diverse environmental sources in the community. J Appl Microbiol 133:1156–1168. doi:10.1111/jam.15408. 34894035

[23] Steyer A, Gutierrez-Aguirre I, Racki N, Beigot Glaser S, Brajer Humar B, Strazar M, Skrjanc I, Poljsak-Prijatelj M, Ravnikar M, Rupnik M. 2015. The detection rate of enteric viruses and *Clostridium difficile* in a waste water treatment plant effluent. Food Environ Virol 7:164–172. doi:10.1007/s12560-015-9183-7. 25663146

[24] Moradigaravand D, Gouliouris T, Ludden C, Reuter S, Jamrozy D, Blane B, Naydenova P, Judge K, Aliyu SH, Hadjirin NF, Holmes MA, Török E, Brown NM, Parkhill J, Peacock S. 2018. Genomic survey of *Clostridium difficile* reservoirs in the East of England implicates environmental contamination of wastewater treatment plants by clinical lineages. Microb Genom 4:e000162. doi:10.1099/mgen.0.000162. 29498619 PMC5885014

[25] Rivas L, Dupont PY, Gilpin BJ, Cornelius AJ. 2020. Isolation and characterization of *Clostridium difficile* from a small survey of wastewater, food and animals in New Zealand. Lett Appl Microbiol 70:29–35. doi:10.1111/lam.13238. 31631350

[26] Romano V, Pasquale V, Krovacek K, Mauri F, Demarta A, Dumontet S. 2012. Toxigenic *Clostridium difficile* PCR ribotypes from wastewater treatment plants in southern Switzerland. Appl Environ Microbiol 78:6643–6646. doi:10.1128/AEM.01379-12. 22798376 PMC3426710

[27] Cizek A, Masarikova M, Mares J, Brajerova M, Krutova M. 2022. Detection of plasmid-mediated resistance to metronidazole in *Clostridioides difficile* from river water. Microbiol Spectr 10:e00806-22. doi:10.1128/spectrum.00806-22. 35950844 PMC9431275

[28] Xu C, Weese JS, Flemming C, Odumeru J, Warriner K. 2014. Fate of *Clostridium difficile* during wastewater treatment and incidence in southern Ontario watersheds. J Appl Microbiol 117:891–904. doi:10.1111/jam.12575. 24930867

[29] Zidaric V, Beigot S, Lapajne S, Rupnik M. 2010. The occurrence and high diversity of *Clostridium difficile* genotypes in rivers. Anaerobe 16:371–375. doi:10.1016/j.anaerobe.2010.06.001. 20541023

[30] Lim SC, Hain-Saunders NMR, Imwattana K, Putsathit P, Collins DA, Riley TV. 2022. Genetically related *Clostridium difficile* from water sources and human CDI cases revealed by whole-genome sequencing. Environ Microbiol 24:1221–1230. doi:10.1111/1462-2920.15821. 34693624

[31] Department of Environment and Conservation. 2012. Western Australian guidelines for biosolids management. Department of Environment and Conservation, Western Australia, Australia. https://www.der.wa.gov.au/component/k2/item/4131-biosolids-management.

[32] Xu C, Wang D, Huber A, Weese SJ, Warriner K. 2016. Persistence of *Clostridium difficile* in wastewater treatment-derived biosolids during land application or windrow composting. J Appl Microbiol 120:312–320. doi:10.1111/jam.13018. 26661445

[33] Dharmasena M, Wei T, Bridges WC, Jiang X. 2019. Thermal resistance of *Clostridium difficile* endospores in dairy compost upon exposure to wet and dry heat treatments. J Appl Microbiol 127:274–283. doi:10.1111/jam.14295. 31034124

[34] Lim SC, Knight DR, Moono P, Foster NF, Riley TV. 2020. *Clostridium difficile* in soil conditioners, mulches and garden mixes with evidence of a clonal relationship with historical food and clinical isolates. Environ Microbiol Rep 12:672–680. doi:10.1111/1758-2229.12889. 32975368

[35] Dharmasena M, Jiang XP. 2018. Isolation of toxigenic *Clostridium difficile* from animal manure and composts being used as biological soil amendments. Appl Environ Microbiol 84:e00738-18. doi:10.1128/AEM.00738-18. 29858208 PMC6070769

[36] Hong S, Putsathit P, George N, Hemphill C, Huntington PG, Korman TM, Kotsanas D, Lahra M, McDougall R, Moore CV, Nimmo GR, Prendergast L, Robson J, Waring L, Wehrhahn MC, Weldhagen GF, Wilson RM, Riley TV, Knight DR. 2020. Laboratory-based surveillance of *Clostridium difficile* infection in Australian healthcare and community settings, 2013–2018. J Clin Microbiol 58:e01552-20. doi:10.1128/JCM.01552-20. PMC7587093 32848038

[37] Knight DR, Squire MM, Riley TV. 2015. Nationwide surveillance study of *Clostridium difficile* in Australian neonatal pigs shows high prevalence and heterogeneity of PCR ribotypes. Appl Environ Microbiol 81:119–123. doi:10.1128/AEM.03032-14. 25326297 PMC4272713

[38] Lim SC, Foster NF, Elliott B, Riley TV. 2018. High prevalence of *Clostridium difficile* on retail root vegetables, Western Australia. J Appl Microbiol 124:585–590. doi:10.1111/jam.13653. 29193458

[39] Moono P, Lim SC, Riley TV. 2017. High prevalence of toxigenic *Clostridium difficile* in public space lawns in Western Australia. Sci Rep 7:41196. doi:10.1038/srep41196. 28145453 PMC5286503

[40] Perumalsamy S, Putsathit P, Riley TV. 2019. High prevalence of *Clostridium difficile* in soil, mulch and lawn samples from the grounds of Western Australian hospitals. Anaerobe 60:102065. doi:10.1016/j.anaerobe.2019.06.018. 31260739

[41] Shivaperumal N, Chang BJ, Riley TV. 2020. High prevalence of *Clostridium difficile* in home gardens in Western Australia. Appl Environ Microbiol 87:e01572-20. doi:10.1128/AEM.01572-20. 33097511 PMC7755239

[42] Knight DR, Kullin B, Androga GO, Barbut F, Eckert C, Johnson S, Spigaglia P, Tateda K, Tsai P-J, Riley TV. 2019. Evolutionary and genomic insights into *Clostridioides difficile* sequence type 11: a diverse zoonotic and antimicrobial-resistant lineage of global One Health importance. mBio 10:e00446-19. doi:10.1128/mBio.00446-19. 30992351 PMC6469969

[43] Numberger D, Riedel T, McEwen G, Nübel U, Frentrup M, Schober I, Bunk B, Spröer C, Overmann J, Grossart H-P, Greenwood AD. 2019. Genomic analysis of three *Clostridioides difficile* isolates from urban water sources. Anaerobe 56:22–26. doi:10.1016/j.anaerobe.2019.01.002. 30633971

[44] Goorhuis A, Bakker D, Corver J, Debast SB, Harmanus C, Notermans DW, Bergwerff AA, Dekker FW, Kuijper EJ. 2008. Emergence of *Clostridium difficile* infection due to a new hypervirulent strain, polymerase chain reaction ribotype 078. Clin Infect Dis 47:1162–1170. doi:10.1086/592257. 18808358

[45] Bauer MP, Notermans DW, van Benthem BHB, Brazier JS, Wilcox MH, Rupnik M, Monnet DL, van Dissel JT, Kuijper EJ, ECDIS Study Group. 2011. *Clostridium difficile* infection in Europe: a hospital-based survey. Lancet 377:63–73. doi:10.1016/S0140-6736(10)61266-4. 21084111

[46] Knight DR, Giglio S, Huntington PG, Korman TM, Kotsanas D, Moore CV, Paterson DL, Prendergast L, Huber CA, Robson J, Waring L, Wehrhahn MC, Weldhagen GF, Wilson RM, Riley TV. 2015. Surveillance for antimicrobial resistance in Australian isolates of *Clostridium difficile*, 2013–14. J Antimicrob Chemother 70:2992–2999. doi:10.1093/jac/dkv220. 26221017

[47] Putsathit P, Hong S, George N, Hemphill C, Huntington PG, Korman TM, Kotsanas D, Lahra M, McDougall R, McGlinchey A, Moore CV, Nimmo GR, Prendergast L, Robson J, Waring L, Wehrhahn MC, Weldhagen GF, Wilson RM, Riley TV, Knight DR. 2021. Antimicrobial resistance surveillance of Clostridioides difficile in Australia, 2015–18. J Antimicrob Chemother 76:1815–1821. doi:10.1093/jac/dkab099. 33895826

[48] Lim SC, Androga GO, Knight DR, Moono P, Foster NF, Riley TV. 2018. Antimicrobial susceptibility of *Clostridium difficile* isolated from food and environmental sources in Western Australia. Int J Antimicrob Agents 52:411–415. doi:10.1016/j.ijantimicag.2018.05.013. 29802886

[49] Collins DA, Riley TV. 2018. *Clostridium difficile* in Asia: opportunities for One Health management. Trop Med Infect Dis 4:7. doi:10.3390/tropicalmed4010007. 30597880 PMC6473466

[50] Water Quality Australia. 2021. Sewage system guidelines. https://www.waterquality.gov.au/guidelines/sewerage-systems. Accessed 20 May 2022.

[51] Government of Western Australia—Department of Health. 2011. Guidelines for the non-potable uses of recycled water in Western Australia. https://ww2.health.wa.gov.au/.

[52] Stubbs SLJ, Brazier JS, O'Neill GL, Duerden BI. 1999. PCR targeted to the 16S-23S rRNA gene intergenic spacer region of *Clostridium difficile* and construction of a library consisting of 116 different PCR ribotypes. J Clin Microbiol 37:461–463. doi:10.1128/JCM.37.2.461-463.1999. 9889244 PMC84342

[53] Clinical and Laboratory Standards Institute. 2011. M11-A7. Methods for antimicrobial susceptibility testing of anaerobic bacteria, seventh edition. CLSI, Wayne, PA. 31339681

[54] Clinical and Laboratory Standards Institute. 2013. M100-S23. Performance standards for antimicrobial susceptibility testing; twenty-third informational supplement. CLSI, Wayne, PA.

[55] The European Committee on Antimicrobial Susceptibility Testing. 2017. Clinical breakpoint tables for interpretation of MICs and zone diameters, version 10.0. EUCAST, European Union. https://www.eucast.org/.

[56] European Medicines Agency. 2011. Assessment report, Dificlir fidaxomicin EMA/857570/2011. EMA, European Union. https://www.ema.europa.eu.

[57] O'Connor JR, Galang MA, Sambol SP, Hecht DW, Vedantam G, Gerding DN, Johnson S. 2008. Rifampin and rifaximin resistance in clinical isolates of *Clostridium difficile*. Antimicrob Agents Chemother 52:2813–2817. doi:10.1128/AAC.00342-08. 18559647 PMC2493101

